# Regional chromatin decompaction in Cornelia de Lange syndrome associated with NIPBL disruption can be uncoupled from cohesin and CTCF

**DOI:** 10.1093/hmg/ddt265

**Published:** 2013-06-10

**Authors:** Leisha D. Nolen, Shelagh Boyle, Morad Ansari, Emily Pritchard, Wendy A. Bickmore

**Affiliations:** MRC Human Genetics Unit, Institute of Genetics and Molecular Medicine, University of Edinburgh, Crewe Road, Edinburgh EH4 2XU, UK

## Abstract

Cornelia de Lange syndrome (CdLS) is a developmental disorder caused by mutations in NIPBL, a protein which has functionally been associated with the cohesin complex. Mutations in core cohesin complex components have also been reported in individuals with CdLS-like phenotypes. In addition to its role in sister chromatid cohesion, cohesin is thought to play a role in regulating gene expression during development. The mechanism of this gene regulation remains unclear, but NIPBL and cohesin have been reported to affect long-range chromosomal interactions, both independently and through interactions with CTCF. We used fluorescence *in situ* hybridization to investigate whether the disruption of NIPBL affects chromosome architecture. We show that cells from CdLS patients exhibit visible chromatin decompaction, that is most pronounced across gene-rich regions of the genome. Cells carrying mutations predicted to have a more severe effect on NIPBL function show more extensive chromatin decompaction than those carrying milder mutations. This cellular phenotype was reproduced in normal cells depleted for NIPBL with siRNA, but was not seen following the knockdown of either the cohesin component SMC3, or CTCF. We conclude that NIPBL has a function in modulating chromatin architecture, particularly for gene-rich areas of the chromosome, that is not dependent on SMC3/cohesin or CTCF, raising the possibility that the aetiology of disorders associated with the mutation of core cohesin components is distinct from that associated with the disruption of NIPBL itself in classical CdLS.

## INTRODUCTION

Cornelia de Lange syndrome (CdLS; OMIM 122470) is a genetic disorder characterized by characteristic facial features, abnormal upper limb development, delayed growth and cognitive retardation (1). These diverse clinical features are indicative of a developmental disorder affecting the expression of multiple genes. Interestingly, all causative mutations identified in cases defined as CdLS have been in genes encoding proteins in the cohesin complex or in proteins that interact with this complex. These include *NIPBL* (2,3), *SMC1A* and *SMC3* (4–7), *RAD21* (8) and *HDAC8* (9). The cohesin complex was initially identified for its role in keeping sister chromatids together during cell division until anaphase; however, recent studies have expanded the role of this complex outside of mitosis and meiosis.

The core components of cohesin are SMC1/SMC3, Scc1/Mcd1/Rad21 and Scc3/stromalin/SA/stag. Together these proteins form a ring-like structure that is responsible for holding sister chromatids together (10). While mutations in genes encoding cohesin complex proteins have been identified in a small subset of patients diagnosed with CdLS, up to 60% of CdLS mutations, and 80% of mutations in the most severe forms of the disease, involve NIPBL (nipped-B-like) which is not a core component of cohesin (3,11–13). NIPBL (Scc2 in *Saccharomyces cerevisiae*) is important for loading the cohesin complex onto chromatin during S-phase (14). Interestingly, another clinical syndrome, Roberts-SC phocomelia, has been found to have mutations in a modulator of cohesin, ESCO2. While sharing some similar clinical characteristics, this syndrome has features that are distinct from CdLS (1), suggesting that subtle changes in the regulation of cohesin function can result in different phenotypes.

Individuals with CdLS are heterozygous for the mutant *NIPBL* allele. Most mutant alleles are predicted to result in either a complete absence of protein or the production of a severely truncated one and thus are considered to be loss of function alleles, though missense mutations have also been reported (3,11,13). Mouse models that are heterozygous for mutant *Nipbl* alleles have some of the phenotypes characteristic of CdLS individuals, including growth retardation, craniofacial abnormalities, heart defects and behavioural changes (15). *NIPBL* mRNA expression levels in both CdLS human cells and mouse models are 60–70% of the normal level, indicating an up-regulation of the wild-type copy (15).

Given that the mutated proteins associated with CdLS are known to be involved in sister chromatid cohesion, one would expect patients to have disorders related to mitosis and meiosis. However, CdLS cell lines do not consistently exhibit premature sister chromatid separation (16) and the phenotypes observed in CdLS individuals suggest a defect in gene regulation rather than in chromatid cohesion during cell division. This may indicate that a single functional copy of *NIPBL* is sufficient to allow chromatid cohesion, but insufficient to perform interphase functions related to developmental gene regulation. Studies of non-dividing cells have demonstrated that components of the cohesin complex do indeed have a role outside of cell division (17–19).

In cell lines from CdLS human patients, as well as cells from CdLS mouse models, a large number of genes are misexpressed at moderate levels when NIPBL is mutated (15,20). The misexpressed genes are more likely to be those that are normally bound by cohesin, and a correlation between disease severity and the degree of change in gene expression was found in CdLS individuals (20). Both of these results support the idea that NIPBL is directly regulating gene expression and that it is the disruption of this function that results in CdLS.

In *Drosophila* Nipped-B, the fly homologue of NIPBL, affects the control of transcription elongation (21) and insulator and enhancer–promoter interactions (18,22–24). These results led to a model in which Nipped-B and the cohesin complex can promote long-range chromatin structures that are involved in bringing enhancers and promoters together to regulate gene expression.

A well-characterized protein involved in DNA looping, insulation and enhancer function is the CCCTC-binding factor (CTCF) zinc finger protein. In mammalian cells, most cohesin-binding sites captured by chromatin immunoprecipitation (ChIP) were first reported to be DNaseI hypersensitive sites also bound by CTCF (25–28). Cohesin is reported to be involved in long-range cis associations detected between CTCF sites at the mammalian *IFNγ*, *APO*, *IgH*, *TCR*, β-globin, HoxA and imprinted gene loci (29–35). However, further studies have shown that NIPBL and cohesin can bind at promoters and enhancers, alongside the Mediator complex, transcription factors and RNA polymerase II (Rpol II), independently of CTCF (36,37).

Given the role of NIPBL and cohesin in long-range chromatin interactions, it is possible that cells from CdLS patients have disrupted higher-order chromatin structure. Here, using interphase fluorescence *in situ* hybridization (FISH) to assay higher-order chromatin compaction (38), we reveal that there is a visible decompaction of chromatin in cells derived from CdLS individuals carrying *NIPBL* mutant alleles. This decompaction is widespread but is most pronounced at specific genomic regions and in cells with the most severe mutations. Chromatin decompaction was also observed in cells depleted of NIPBL by siRNA. Genomic regions with a high gene density were more likely to be decompacted in both patient cells and in NIPBL knockdown cells than gene-poor regions. Surprisingly, SMC3 knockdown resulted in detectable chromatin decompaction at only one of the tested loci and not at the loci that were most susceptible to NIPBL mutation or knockdown. Moreover, the knockdown of CTCF did not result in visible chromatin decompaction at any of the tested loci.

We conclude that NIPBL plays an important role in chromatin compaction, having the most impact in regions of the genome with the highest gene density and the highest density of CTCF and cohesin binding sites. We suggest that the effects on chromatin compaction are independent of CTCF and indeed also largely independent of SMC3. This raises the possibility that the clinical phenotypes of CdLS individuals with NIPBL mutation might result from the perturbation of a function of NIPBL that is independent of its role in cohesion or cohesin biology.

## RESULTS

### Nuclear size in CdLS cell lines

To determine whether there are global changes in chromatin compaction associated with the mutation of *NIPBL*, we first analysed nuclear size in fixed lymphoblastoid cell lines (LCLs) from individuals with a severe CdLS phenotype. Many factors affect nuclear size within a particular cell type, but chromatin decompaction is known to result in nuclear swelling (39–41).

**Figure 1.**
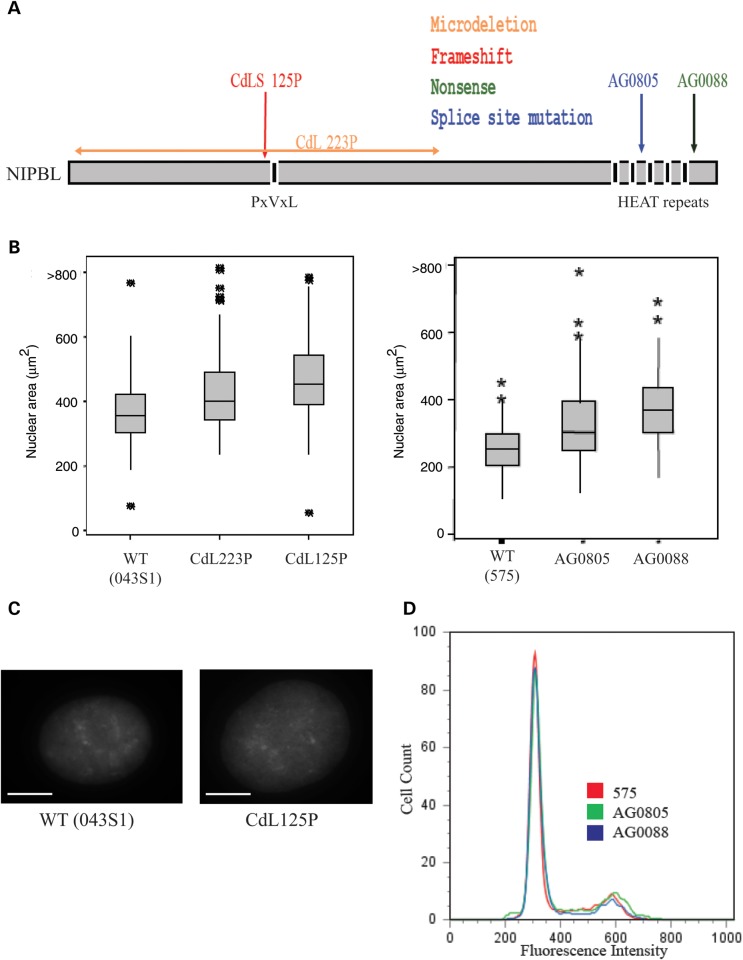
Characterization of NIPBL cell lines. (**A**) Representation of NIPBL protein with the main protein domains shown below and the location of CdLS mutations in LCLs indicated above. Orange represents a microdeletion, red a frameshift, blue a splice site mutation and green a nonsense mutation. (**B**) Box plots showing the distribution of nuclear sizes (μm^2^) in fixed nuclei from control and CdLS LCLs. Data from CdLS lines CdL223P and CdL125 are shown on the left together with a wild-type unaffected sibling (WT). AGO805 and AG0088 are shown on the right together with an independent WT control (575). Horizontal lines show the means and the boxed area is the interquartile range (IQR). Whiskers show 1.5 IQR of the upper and lower quartile. *n* > 100 loci each, *P* < 0.001. (**C**) Sample images of DAPI-stained nuclei from wild-type and patient cells demonstrating differences in nuclear size. Scale bar = 10 μm. (**D**) Fluorescence-activated cell sorting (FACS) analysis of DNA content from PI stained WT (575), AG0805 and AG0088 cells.

Cell lines were chosen that carry different types of mutations in *NIPBL* with predicted differing effects on the resulting protein*.* CdL223P carries an *NIPBL* allele with a deletion of exons 2–17 that removes the start codon for NIPBL (Fig. 1A and Table 1) (42). CdL125P carries an allele with a very early frameshift (11) that is the most common mutation found in CdLS (43) and is expected to create no, or a severely truncated, protein. Two other cell lines have point mutations within (AG0805), or just after (AG0088), the conserved C-terminal HEAT domains (2), which have been shown to be necessary to recruit NIPBL to sites of DNA damage (44) as well as mediating protein–protein interactions (45,46). By imaging 4′,6-diamidino-2-phenylindole (DAPI) stained fixed nuclei, we found that CdLS cell lines had a significant increase in nuclear size distribution relative to wild-type LCLs (*P* < 0.001; Fig. 1B and C).

**Table 1.** CdLS cell lines

Cell line	Mutation type	Location of mutation in *NIPBL*	Amino acid change in NIPBL	Karyotype	Reference
CdL 223 P	Microdeletion	Deletion of exons 2–17	No start codon	46XX	(42)
CdL 125 P	Frameshift	2479_2480delAG	R827GfsX2	50% 46XX, 50% 44XX + fusion (poss 1;16)	(11,43)
AG0088	Frameshift	7306_7307 ins G	S2435X	46XY	(2)
AG0805	Splice site	5575–2 A > G	No exon 30	46XX	(2)

The type of mutation in *NIPBL* in the four CdLS LCL cell lines studied is indicated as is the predicted effect on NIPBL protein. Patient phenotypes were reported in the indicated references. Karyotypes for three of the four cell lines were normal. For CdL125P cell line, 50% of metaphases were normal 46XX but in 50% of cells there was a fusion chromosome. This did not appear to involve any of the regions analysed in this paper.

Nuclear size increases during the cell cycle, so one possibility was that CdLS cell lines have an altered cell cycle profile, perhaps resulting from aberrant sister chromatid cohesion. However, fluorescence-activated cell sorting (FACS) analysis showed that the CdLS LCLs have a normal cell cycle distribution relative to control LCLs (Fig. 1D). Therefore, we conclude that the increased nuclear size we observe in CdLS cells is not the result of abnormal cell cycle dynamics and may instead reflect altered chromatin compaction.

### Regional chromatin decompaction in CdLS cell lines correlates with NIPBL mutation

**Figure 2.**
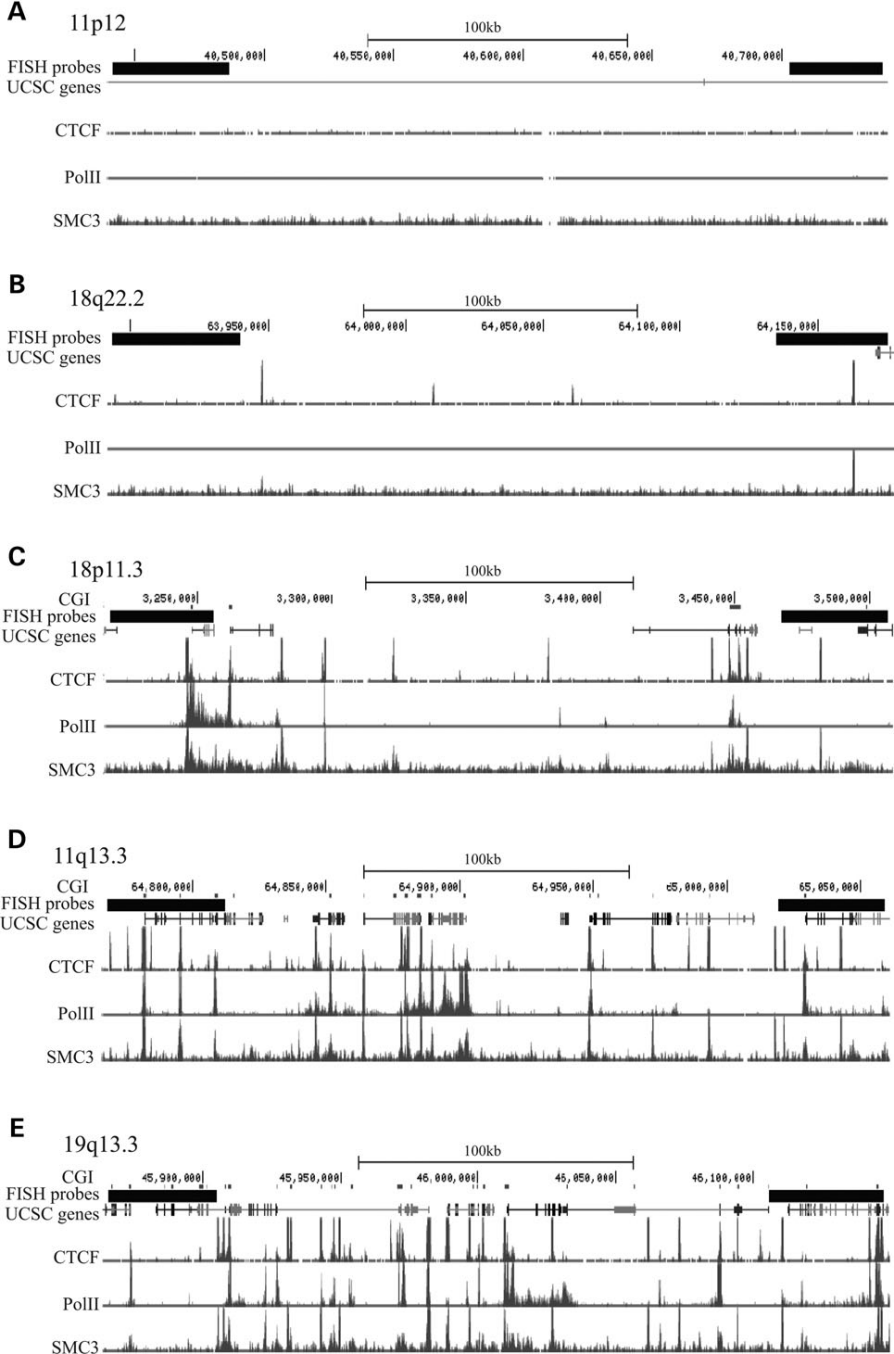
Genomic regions used for FISH analyses. UCSC genome browser images showing the location of FISH probes in the five tested genomic regions. Map position (Mb) and RefSeq gene annotations are from the February 2009 (hg19) assembly of the human genome (http://genome.ucsc.edu). Positions of UCSC genes and CGIs are also indicated. ChIP peaks for CTCF, Rpol II and SMC3 are from ENCODE data for GM12878 LCLs. (**A**) Chromosome 11p12 region with low gene density. (**B**) Chromosome 18q22.2 region with low gene density. (**C**) Chromosome 18p11.3 region with moderate gene density. (**D**) Chromosome 11q13.3 region with high gene density. (**E**) Chromosome 19q13.3 region with high gene density.

To determine whether NIPBL mutations affect higher-order chromatin compaction at particular loci, we selected specific regions of the human genome to examine in more detail. The five regions were chosen to represent a variety of different genomic characteristics (Table 2). Because of the reported binding of NIPBL at gene promoters in association with Rpol II and Mediator (36), we selected two regions of low gene and CpG island (CGI) density, 11p12 and 18q22.2 (Fig. 2A and B), one of moderate gene density, 18p11.3 (Fig. 2C), and two regions, 11q13.3 and 19q13.3, of high gene and CGI density (Fig. 2D and E). Moreover, 11q13.3 is also a region of the human genome (RIDGEs) where densely packed genes are also expressed to a high level (47).

**Table 2.** Characteristics of Fosmid probe locations

Chromosome	Whitehead probe name	Other probe name	Start (bp)	End	Midpoint	Separation between probe pair midpoints (bp)	Genes/100 kb	CGI/100 kb	CTCF/100 kb	SMC3/100 kb
11p12	WI2-1843D17	G248P86589B9	40 702 638	40 738 514	40 720 576	256 605.0	<1	0	0	0
	WI2-0676N14	G248P80235G7	40 441 561	40 486 381	40 463 971					
18q22.2	WI2-1702P7	G248P87869H4	64 134 988	64 175 629	64 155 309	238 833.5	0	0	1.6	1
	WI2-502C21	G248P8988B11	63 893 119	63 939 831	63 916 475					
18p11.3	WI2-0672M24	G248P80018G12	3 467 167	3 506 775	3 486 971	250 195.0	3	1.5	5.5	3.5
	WI2-1795M06	G248P86030G3	3 217 757	3 255 795	3 236 776					
11q13.3	WI2-671I21	G248P80020E11	64 768 160	64 811 897	64 790 029	249 082.0	9	9	9	10
	WI2-1737E8	G248P86034C4	65 019 283	65 058 937	65 039 110					
19q13.3	WI2-1832N17	G248P86553G9	46 105 895	46 147 486	46 126 691	241 287.0	7	7	12	13
	WI2-1336P19	G248P84001H10	45 865 675	45 905 133	45 885 404					

Probe names are from the Whitehead Fosmid database (http://bacpac.chori.org/library.php?Id=275). Alternative probe names can be used to view fosmids on the UCSC genome browser. All genome locations are reported as hg19 coordinates. Gene, CGI, CTCF and SMC3 peak densities are estimates based on UCSC genome browser and Encode datasets and averaged for a 100 kb region.

Rpol II binding was also ascertained from the ChIP data for LCLs established by ENCODE (48). No Rpol II binding is detected at either of the low gene-density regions, while peaks of binding are found scattered throughout the other three regions. In agreement with this, gene expression data from human LCLs show transcripts originating from the high and moderate gene-density regions, while no transcripts are detected in the regions of low gene density (49).

The selected regions also have different CTCF binding profiles that largely follow the trends in gene density consistent with the location of many CTCF sites close to the transcription start sites of genes (50,51). Finally, and as expected given the correlation between CTCF and cohesin subunit ChIP signals (25–28), cohesin density (SMC3 ChIP peaks) also varies across the selected genomic regions in line with gene density (Fig. 2 and Table 2).

We assayed higher-order chromatin compaction in control and CdLS LCLs at these five selected genomic regions by FISH using probe pairs separated by 250 kb (Fig. 2 and Table 2). The mean-squared interprobe distance (*d*^2^) between hybridization signals for such probe pairs is known to have a linear relationship with genomic separation (kb) over this size range and can be used to measure changes in chromatin compaction both between different regions of the same genome (52,53) and between different cell types—e.g. during differentiation and development (54,55). Moreover, such analysis can determine the role of specific histone modifications and proteins in chromatin compaction in wild-type and mutant cells (38). In order to detect effects that are locus-specific as opposed to those that are just a reflection of genome-wide chromatin decompaction and increased nuclear size, data were normalized to the nuclear radius (*r*^2^), as described previously (38).

**Figure 3.**
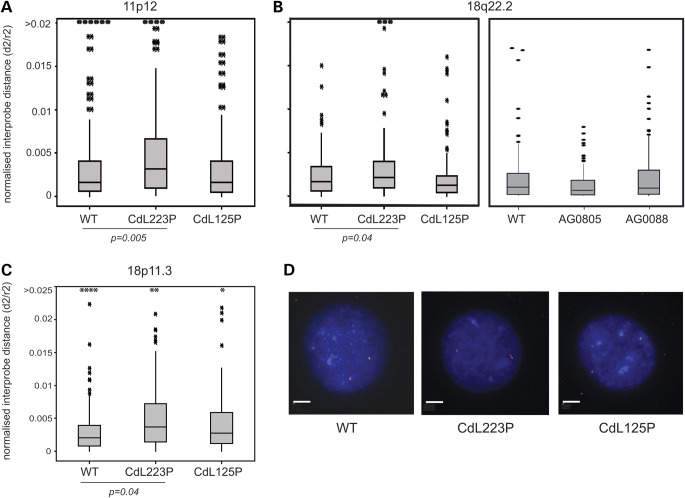
Chromatin compaction at gene-poor regions in CdLS. Box plots show the distribution of interprobe distances^2^ (*d*^2^) normalized for nuclear radius^2^ (*r*^2^) in FISH across 250 kb regions in unaffected and CdLS patient cell lines. The shaded boxes show the median and interquartile range of the data; asterisks indicate outliers. The statistical significance of differences between unaffected sibling and the CdLS cell lines were determined by the Mann–Whitney *U*-tests. All *P*-values are reported in relation to unaffected cells in Table 3 and significant values are indicated below the graphs. (**A**) 11p12 gene and CTCF low region, *n* > 95. (**B**) 18q22.2 gene and CTCF low region, *n* > 97. (**C**) 18p11.3 region with moderate gene and CTCF density, *n* > 87. (**D**) Example FISH images from WT, CdL223P and CdL125P cells for the 11p12 region. Bar represents 5 µm.

FISH analysis at regions of low to moderate gene density (Fig. 3) showed that in three of the four CdLS cell lines, there was no significant change (*P* > 0.05, Table 3) in regional chromatin compaction compared with wild-type cells. There was also no altered chromatin compaction detected at the 18q22 locus in CdLS lines AG0805 and AG0088 with a probe pair separated by a larger (400 kb) genomic distance (data not shown). The only cell line where there was a significant decrease in regional chromatin compaction (increase in *d*^2^/*r*^2^) in regions of low gene density, relative to control cells, was CdL223P, the cell line that is expected to have the most profound impairment of NIPBL function.

**Table 3.** Statistical results from FISH analysis of CdLS cell lines

	11p12	18q22.2	18p11.3	11q1.3	19q13.3
*Normalized interprobe distance (d^2^/r^2^)*					
Unaffected sibling	0.00162	0.00161	0.00206	0.00295	0.00206
CdL223P	0.00324	0.0022	0.00365	0.00713	0.00758
	*P* = 0.0054	*P* = 0.0388	*P* = 0.0031	*P* = 0.0006	*P* < 0.0001
CdL125P	0.00155	0.00122	0.00278	0.00552	0.00575
	*P* = 0.8593	*P* = 0.0822	*P* = 0.1022	*P* = 0.0085	*P* < 0.0001
WT (575)		0.002		0.0036	
AG0805		0.0013		0.0043	
		*P* = 0.688		*P* = 0.007	
AG0088		0.002		0.0035	
		*P* = 0.787		*P* = 0.178	
*Squared interprobe distance (d^2^)*					
Unaffected sibling	0.1698	0.1782	0.1991	0.3305	0.1991
CdLS 223P	0.3548	0.1726	0.4181	0.7442	0.7598
	*P* = 0.0014	*P* = 0.7038	*P* = 0.0001	*P* = 0.0016	*P* < 0.0001
CdLS patient 125	0.2003	0.1251	0.3429	0.8425	0.8723
	*P* = 0.4797	*P* = 0.1336	*P* = 0.0035	*P* < 0.0001	*P* < 0.0001
WT (575)		0.15		0.41	
AG0805		0.25		1.04	
		*P* = 0.118		*P* = 0.001	
AG0088		0.16		0.49	
		*P* = 0.495		*P* = 0.697	

Mean normalized distance (*d*^2^/*r*^2^) and squared interprobe distances (*d*^2^) for FISH data from control and CdLS LCLs at five genomic loci, using probe pairs separated by 250 kb. *P*-values were generated using the Mann–Whitney *U* non-parametric test comparing CdLS to wild-type controls. Significant *P*-values (<0.05) are indicated by shaded boxes.

**Figure 4.**
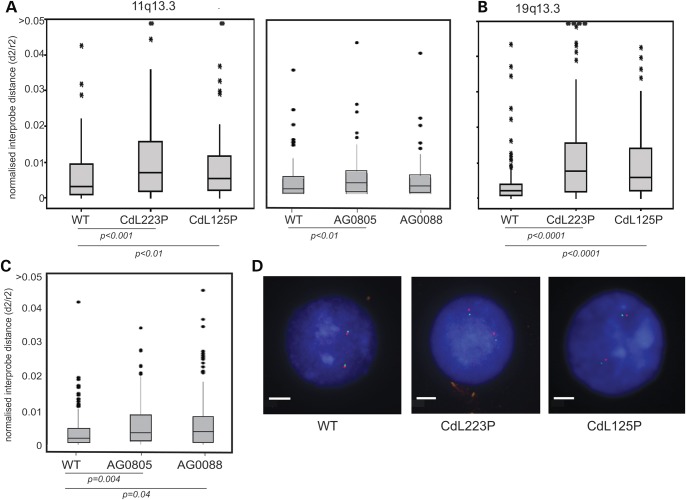
Chromatin compaction at gene-rich regions in CdLS. As in Figure 3 but for (**A**) 11q13.3 gene- and CTCF-rich region, *n* > 100 and (**B**) 19q13.3 gene- and CTCF-rich region, *n* > 95. All *P*-values are reported in relation to unaffected sibling in Table 3 and significant values are indicated below the graphs. (**C**) 11q13.3 gene- and CTCF-rich region, assayed with probes separated by 400 kb, *n* > 100. (**D**) Example FISH images from WT, CdL223P and CdL125P cells for 19q13.3. Bar represents 5 µm.

In contrast, at the two regions of high gene and high CTCF/SMC3 density, significant chromatin decompaction was seen across the 250 kb size range in both CdL223P and CdL125P cell lines (Fig. 4A, B and D). Moreover, for CdL223P cells, the extent of decompaction measured at these loci relative to wild-type cells was greater (2.4-fold for 11q13.3 and 3.7-fold for 19q13.3) than that at the regions of moderate to low gene density (<2-fold; Table 3). The 11q13.3 region was also analysed in AG0805 and AG0088 CdLS cell lines (Fig. 4A). Significant chromatin decompaction was seen in AG0805, but not in AG0088 which potentially has the mildest mutation, a nonsense mutation at the extreme C-terminus of NIPBL (Fig. 1A). Significant chromatin decompaction was, however, seen in AG0088 cells when the probe pair separation was increased to 500 kb (Fig. 4C).

To exclude that the increased nuclear distances seen by FISH in CdLS patients could be due to a genomic alteration, e.g. copy number variation, in the regions being tested, we used array comparative genome hybridization (array CGH) on genomic DNAs prepared from AG0805, AG0088 and CdL125P. The results showed no indication of gross genomic rearrangements at a genome-wide level in any of the three samples analysed. Detailed inspection of the regions examined by FISH revealed no significant changes in genomic copy number in those regions (Supplementary Material, Fig. S1).

From our data, we conclude that loss of NIPBL function causes a large-scale unfolding of higher-order chromatin structure that can be detected by FISH and that this occurs to the greatest extent in genomic regions with the highest gene density and the highest density of CTCF and cohesin binding sites. Our data also suggest that different mutations of *NIPBL* impact on the extent of chromatin decompaction, with the most severe cellular phenotype seen in the cases where the mutation is predicted to have the most severe effect on NIPBL protein and protein function.

### Chromatin decompaction after knockdown of *NIPBL*

**Figure 5.**
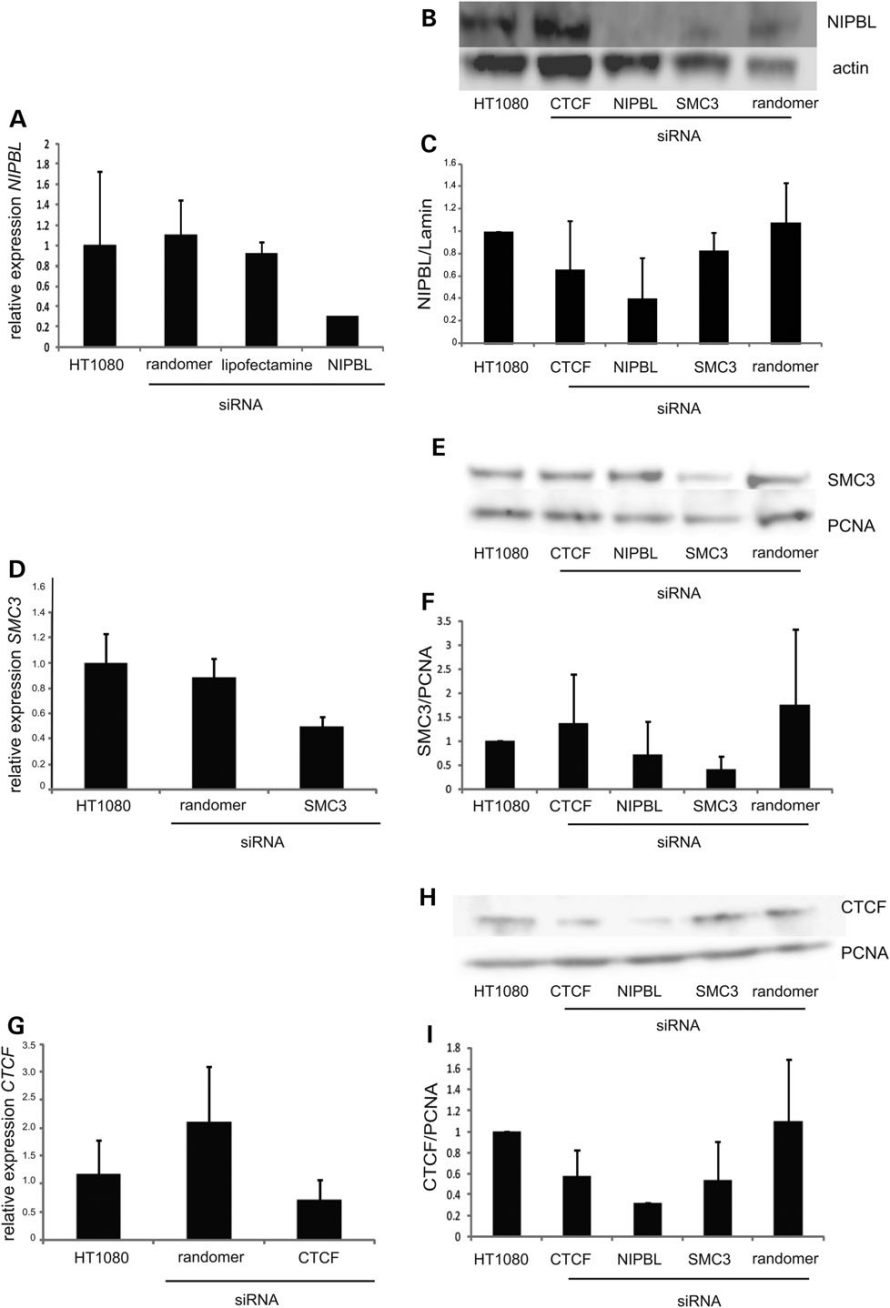
Knockdown of NIPBL, SMC3 and CTCF. (**A**) Real-time quantification of *NIPBL* mRNA levels relative to those for the mRNA of NADH dehydrogenase (ubiquinone) 1 alpha (*NDUFA1*) in untreated cells, in mock transfected cells and in cells transfected with random siRNAs or with NIPBL-specific siRNA. All values are normalized to levels in untreated cells (value of 1). All analysis performed in triplicate. (**B**) Western blot for NIPBL and actin in untreated cells and in cells after siRNA knockdown for CTCF, NIPBL and SMC3. Data for transfection with a random siRNA control are also shown. (**C**) Quantification of NIPBL protein levels based on western results. Samples normalized with actin levels and relative to the ratio in untreated cells. (**D**) As in (A) but for *SMC3* knockdown. (**E**) As in (B) but for SMC3 and PCNA. (**F**) As in C but SMC3 protein levels normalized to those of PCNA. (**G**, **H** and **I**) As in (D), (E) and (F) but for CTCF knockdown.

Because NIPBL expression levels are reported to be reduced to only 60–70% of wild-type levels in CdLS (15,20), we analysed the effects on chromatin compaction of more severely reduced levels of NIPBL, achieved by siRNA knockdown in HT1080 human fibrosarcoma cells. Knockdown of NIPBL at both the mRNA and the protein level was confirmed by quantitative reverse transcription PCR (rtPCR) and western blot (Fig. 5A and B). We estimate that NIPBL protein and mRNA levels are reduced to ∼20% of those in untreated cells (Fig. 5A and C).

**Figure 6.**
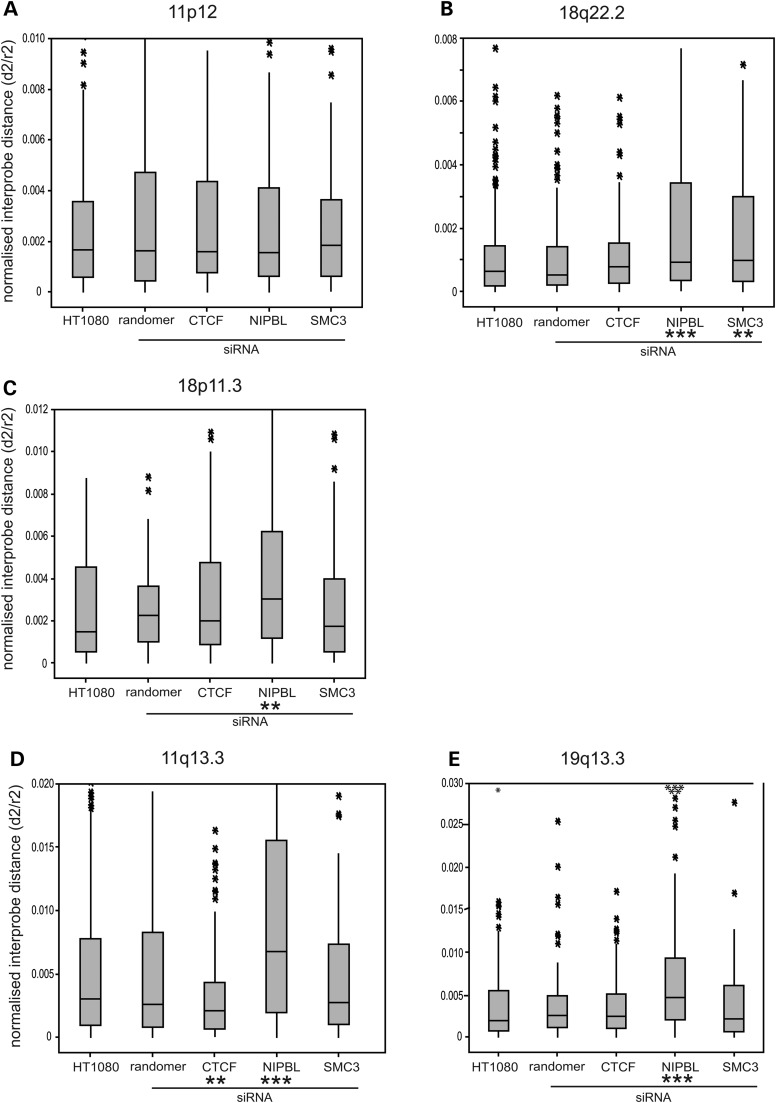
Regional chromatin compaction after NIPBL, SMC3 and CTCF knockdown. Box plots show the distribution of interprobe distances^2^ (*d*^2^) normalized for nuclear radius^2^ (*r*^2^) in untreated cells and after knockdown with siRNAs against NIPBL, SMC3, CTCF and with random siRNAs. All *P*-values are reported in relation to untreated cells (Table 4) and are indicated here by *P < 0.05 and >0.01, **P < 0.01 and >0.001 and ***P < 0.001 and >0.0001. Analysis at (**A**) 11p12 gene- and CTCF-poor region, *n* > 105; (**B**) 18q22.2 gene- and CTCF-poor region, *n* > 100; (**C**) 18p11.3 region with moderate gene and CTCF density, *n* > 110; (**D**) 11q13.3 gene- and CTCF-rich region, *n* > 100; (**E**) 19q13.3 gene- and CTCF-rich region, *n* > 100.

FISH was then performed on these cells to assess the consequence of NIPBL knockdown on chromatin compaction at the same five specific genomic regions that had been examined in CdLS cells. At the gene/CTCF/cohesin poor region on chromosome 11 (Fig. 2A), there was no detectable change in relative chromatin compaction in NIPBL knockdown cells compared with either untransfected cells or a random siRNA control (Fig. 6A and Table 4).

**Table 4.** Statistical results for FISH analysis of siRNA knockdown of NIPBL, SMC3 and CTCF

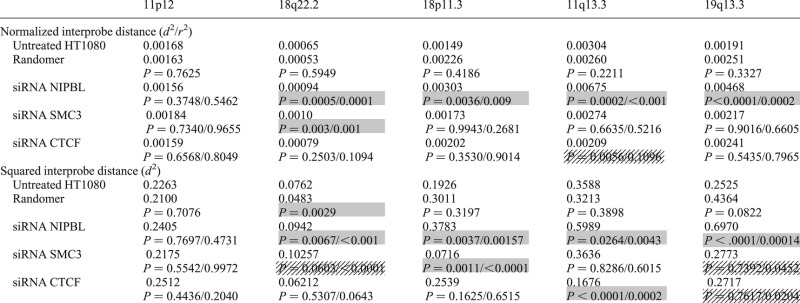					

Mean normalized distance (*d*^2^/*r*^2^) and squared interprobe distances (*d*^2^) for FISH data from untreated HT1080 cells and from cells transfected with random siRNAs or siRNAs specific for NIPBL, SMC3 or CTCF. FISH was conducted at five genomic loci, using probe pairs separated by 250 kb. *P*-values were generated using the Mann–Whitney *U* non-parametric test comparing specific siRNA knockdown to untreated (first value) or to random siRNA (second value). Significant *P*-values (<0.05) are indicated by shaded boxes. If *P*-values are significant for only comparison to untreated, or control siRNA, but not both, then boxes are stippled.

However, at all other loci tested, significant chromatin decompaction was seen after NIPBL knockdown compared with either the untreated cells or the random siRNA control (Fig. 6B–E and Table 4). As in CdLS cells, the extent of chromatin decompaction after NIPBL knockdown was greater at the gene-dense regions than at gene poor loci.

### Absence of chromatin decompaction after the knockdown of *CTCF* and *SMC3*

NIPBL could exert its effect on chromatin compaction through its role in cohesin loading. This would be consistent with the fact that some cases diagnosed as CdLS are associated with mutations in the cohesin complex itself. In order to evaluate the role of other components of the cohesin complex in chromatin compaction, we knocked down SMC3 in the HT1080 cells. Knockdown was confirmed by rtPCR and western blot (Fig. 5D–F). Expression of SMC3 was reduced to 55% the level in untreated cells after specific siRNA treatment.

FISH on SMC3 knockdown cells did not reveal the same extensive chromatin decompaction across the tested genomic regions as was seen with NIPBL knockdown (Fig. 6 and Table 4). This is particularly significant since SMC3 knockdown did appear to reduce NIPBL protein levels to a small degree (Fig. 5C). There was a small effect (<2-fold) on chromatin compaction at 18q22.2 after SMC3 knockdown, although paradoxically there are no annotated SMC3/cohesin binding sites at this genomic region (Fig. 2B), but there was no chromatin decompaction detected at the gene-dense regions with the highest density of SMC3 ChIP peaks and which are the regions also most affected by NIPBL knockdown (Fig. 2D and E). This suggests that the effects of NIPBL on chromatin compaction may be independent of the cohesin complex itself.

Since many NIPBL sites have been reported to be coincident with CTCF binding sites (25–28) and CTCF has been suggested to affect higher-order chromatin conformation through chromatin compaction (32) and ‘looping’ (56,57), we also assayed higher-order chromatin compaction after CTCF knockdown by siRNA. CTCF RNA and protein levels were reduced to ∼60% of wild-type (Fig. 5G–I).

The only significant change in chromatin compaction detected by FISH after CTCF knockdown was at the 11q13 locus, and in fact this was a decrease in *d*^2^/*r*^2^ and *d*^2^ values compared with untreated cells (Table 4), i.e. an increase in chromatin compaction. Although NIPBL knockdown appears to significantly reduce levels of CTCF as well as NIPBL (Fig. 5I), given the absence of detectable chromatin decompaction upon CTCF knockdown, we conclude that the decompaction of chromatin seen in NIPBL depleted or NIPBL mutant cells is attributable to NIPBL itself and is not due to the association of NIPBL with a cohesin component (SMC3) or with CTCF.

## DISCUSSION

The most common gene mutated in individuals diagnosed as CdLS is *NIPBL*. However, the link between the genotype of these individuals and their biological phenotypes remains unclear. It is accepted that the CdLS clinical phenotypes associated with NIPBL mutation are not the result of mitotic/sister chromatid cohesion defects, but instead result from the altered regulation of gene expression. This is consistent with the reported genomic localization of NIPBL with components of the transcriptional machinery (36). How perturbation of NIPBL affects gene expression has not been established.

Here, we have demonstrated that there is a decompaction of higher-order chromatin structure at a subset of genomic regions in cells from CdLS individuals and that this is seen to the greatest degree at regions that are gene-rich and characterized by a high density of binding sites for cohesin and CTCF as assessed by ChIP (Figs 3 and 4). We provide evidence that the extent to which chromatin compaction is perturbed is linked to the likely severity of the NIPBL mutation, and this is further supported by the extensive chromatin decompaction we report after NIPBL knockdown by siRNA (Figs 5 and 6). These observations are consistent with the chromatin decondensation seen in budding yeast with mutations analogous to CdLS mutations, in *Scc2*—the yeast homologue of *NIPBL* (58).

Given that both NIPBL and cohesin affect long-range chromatin interactions in genetic assays, and cohesin affects chromatin conformation as measured in 3C cross-linking assays (29,30,32), we evaluated chromatin compaction in cells after the knockdown of the cohesin component SMC3. This did not phenocopy the effect seen either with NIPBL knockdown or in CdLS-associated NIPBL mutant cells.

Despite the fact that many NIPBL sites appear coincident with CTCF binding sites (25–28) and CTCF has been suggested to affect higher-order chromatin conformation (32,56,57), we also did not see chromatin decompaction after CTCF knockdown, even at genomic regions with the highest density of CTCF binding sites. We note that we were unable to decrease the levels of CTCF below 60% by siRNA treatment—CTCF is required for cell viability and division and is notoriously hard to knockdown (59). However, we did paradoxically observe an increase in chromatin compaction at the 11q13.3 locus after CTCF knockdown, indicating that knockdown was significant enough to have an impact on chromatin structure, albeit not in the expected direction.

Our data, therefore, suggest that NIPBL can affect higher-order chromatin folding, independent of cohesin and CTCF. This raises the possibility that the underlying aetiology of disorders associated with the mutation of core cohesin components is different from that associated with the disruption of NIPBL. Individuals identified with mutations of SMC1A and SMC3 have relatively mild phenotypes, often with atypical facial features and fewer limb and digit abnormalities than those which characterize classic CdLS with *NIPBL* mutation (6,13,43). This has been attributed to the fact that the SMC1A/SMC3 mutations have a predicted modest affect on protein structure—more severe mutations are assumed to be incompatible with birth—but it could also reflect a fundamental difference in the cellular functions of NIPBL and cohesin and a different aetiology for NIPBL-associated CdLS and the CdLS-like diseases associated with mutations in cohesin components. This would be consistent with the differences in dysregulated gene expression seen in zebrafish morphants for nipbl and those depleted in cohesin components (60).

Future work to dissect the regional differences in susceptibility to chromatin decompaction associated with NIPBL mutation, to establish how this level of chromatin structure is linked to the disruption of gene expression and to examine this in different cell types and at different developmental stages in animal models of CdLS has the potential to provide new insight into the mechanisms of CdLS-like diseases.

## MATERIALS AND METHODS

### Cell culture

LCLs were cultured in RPMI-1640 supplemented with 0.2 mm l-glutamine, 100 U/ml penicillin, 100 μg/ml streptomycin and 20% fetal calf serum (FCS). HT1080 fibrosarcomma cells (61) were cultured in Dulbecco's modified Eagle medium (Invitrogen) supplemented with 0.2 mm l-glutamine, 100 U/ml penicillin, 100 μg/ml streptomycin and 10% FCS. For cell cycle analysis, harvested cells were resuspended in 50% FCS/phosphate buffered saline (PBS), 3× volume 70% ice-cold ethanol was added and cells were stored at 4°C. Cells were then washed with PBS and resuspended in propidium iodide solution (50 µg/ml PI + 100 µg/ml RNAse) for 1 h prior to FACS analysis on a BD FACSAria2 SORP. Cells were excited at 488 nm and measured at 562–588 nm. Data were analysed using BD FACSDiva Software version 6.1.3.

### FISH, image capture and analysis

Nuclei were isolated in hypotonic buffer (0.25% KCl) and fixed with 3:1 v/v methanol/acetic acid. Fosmid clones (Table 2) were prepared and labelled with digoxigenin-11-dUTP or with biotin-16-dUTP as described previously (55). After hybridization, digoxigenin-labelled probes were detected using Rhodamine anti-digoxigenin and Texas Red anti-sheep IgG (Vector Laboratories). Biotin-labelled probes were detected using fluorescein–streptavidin and biotinylated anti-avidin (Vector Laboratories).

Slides were analysed as described previously (55) except that a Chroma #83000 triple band pass filter set (Chroma Technology Corporation, Rockingham, VT, USA) and a motorized filter wheel (Prior Scientific Instruments, Cambridge, UK) were used.

### Array CGH

Genomic DNA was prepared from LCLs using a Nucleon DNA extraction kit (Tepnel Life Sciences, UK). DNA was quantified by NanoDrop spectrophotometry (Thermo Scientific).

Genome-wide analysis of DNA copy number aberrations was carried out using the Roche Nimblegen 135k whole-genome array (median probe spacing of ∼12 kb) according to the manufacturer's instructions, as described previously (62). After washing, slides were scanned using the Roche MS200 scanner and analysed using NimbleScan software (Roche Nimblegen). The CGH-segMNT module of NimbleScan was used for the analysis with a minimum segment length of 5 probes and an averaging window of 130 kb. The results were analysed by obtaining the log_2_ ratios of the case (labelled with Cy-5) compared with the control (labelled with Cy-3). A heterozygous deletion and a heterozygous duplication are expected to result in log_2_ ratios of −1.0 and 0.58, respectively. Similarly, a homozygous deletion and a homozygous duplication are expected to result in log_2_ ratios of −2.0 and 1.0, respectively. Genomic coordinates were converted from hg19 to hg18 for analysis, using the UCSC Lift Genome Annotations utility.

### SiRNA knockdown

SiRNA-mediated knockdown was performed using On-Targetplus SMARTpool siRNA mixes (Thermo Scientific), designed with dual-strand modification that decreases off target effects and containing a mix of four individual siRNAs Table 5). The randomer was the ON-TARGET*plus* Non-targeting Control Pool. siRNAs were diluted as directed by the manufacturer to a stock concentration of 100 µm in the supplied 1× siRNA buffer. A 10 cm dish of HT1080 cells at 90% confluence was lipofected with 165 pmol siRNAs using Lipofectamine 2000. Cells were harvested for RNA and protein 50 h post-transfection.

**Table 6.** Primers for real-time rtPCR

Primer name	Primer sequence	Product size
CTCFrealF	TGACACAGTCATAGCCCGAAAA	74
CTCFrealR	TGCCTTGCTCAATATAGGAATGC	
NIPBL2F	GGAGTGACATGGCTAATTCC	103
NIPBL2R	TCATCAGGGTCCAGATGTTC	
SMC3F	TTGACCAGGCTCTGGATG	250
SMC3R	CTCCCAAACCAGTAGGTAG	
NDUFA1F	ACTGGCTACTGCGTACATCC	104
NDUFA1R	AGATGCGCCTATCTCTTTCC

**Table 5.** Sequences of On-Targetplus SMARTpool siRNA

SMC3	gcagugcaacacagaauua	gagaguagaugcacugaau	gguguaaaguucagaaaua	caacguagcuuacagaguu
CTCF	gaacagcccauaaacauag	gaagaugccugccacuuac	ggagaaacgaagaagagua	gaugaagacugaaguaaug
NIPBL	gggcuuguuucaauagaua	acacuucacuucuaacaaa	caacagaucacauagaguu	gauauaaaccgcccacuaa

### Real-time PCR

RNA was isolated using Qiagen RNA Easy Kit. 500 ng of RNA were reverse transcribed in a 20 µl reaction using Superscript II. 1 μl of cDNA was amplified using specific primers (Table 6) and SYBER green real-time master mix (Life Technologies). Real-time analysis was performed using a Roche LightCycler 480. The programme for amplification was 95°C × 3 min, then 45 cycles of 95°C × 15 s, 62^o^C × 15 s and 72^o^C × 30 s. The melting curve programme was 95°C × 5 s and 65°C × 1 min, and then readings were acquired while increasing the temperature by 0.11°C/s to 97°C.

### Immunoblotting

Protein was isolated from a 6-well plate of cells. 100 μl of 4× SDSLB (2.0 ml 1 m Tris–HCl, pH 6.8, 0.8 g sodium dodecyl sulphate SDS), 4.0 ml of 100% glycerol, 0.4 ml of 14.7 m β-mercaptoethanol, 1.0 ml of 0.5 m ethylenediaminetetraacetic acid 8 mg bromophenol blue) and 300 µl PBS were added to the cells. The cells and liquid were collected from the wells, boiled for 5 min and then sonicated for 20 s (Bioruptor NextGen Diagenode) prior to loading directly onto gels. For analysis of NIPBL, samples were run on a NuPage TrisAcetate 3–8% gel (Life Technologies) using the NuPage running buffer. For CTCF and SMC3, samples were run on a standard 10% SDS polyacrylamide gel. Gels were transferred onto polyvinylidene fluoride membranes by semidry transfer at 10 V for 1 h. The membrane was blocked for 1 h in 5% milk PBST (0.1% Tween-20 in Dulbecco A PBS; Oxoid). NIPBL was detected with the anti-IND3 rat monoclonal antibody (AbCAM #131913) diluted 1:500. For Smc3, anti-SMC3 rabbit polyclonal antibody (Bethyl A300-060A) was used at a 1:1000 dilution. For CTCF, anti-CTCF rabbit polyclonal antibody (Upstate 07-729) was used at a 1:2000 dilution. Membranes were incubated with antibodies overnight in 1.5% milk PBST at 4°C, washed 3× with PBST and incubated with the appropriate horseradish peroxidase-conjugated secondary antibody at a 1:10 000 dilution at room temperature for 1 h. Signal was detected by ChemiGlow West (Alpha Innotech) and imaged using Image Quant LAS4010 (Version 1, Build 1.0.0.52; GE Healthcare).

## SUPPLEMENTARY MATERIAL

Supplementary Material is available at *HMG* online.

## FUNDING

This work was supported by an EMBO long-term fellowship and a Medical Research Council Centenary award to L.D.N. and a PhD studentship from the Medical Research Council to E.P. W.A.B. is supported by the Medical Research Council and by an ERC advanced grant (249956). Funding to pay the Open Access publication charges for this article was provided by Medical Research Council UK.

## Supplementary Material

Supplementary Data

## References

[1] Liu J., Krantz I.D. (2008). Cohesin and human disease. Ann. Rev. Genomics Hum. Genet..

[2] Tonkin E.T., Wang T.J., Lisgo S., Bamshad M.J., Strachan T. (2004). NIPBL, encoding a homolog of fungal Scc2-type sister chromatid cohesion proteins and fly Nipped-B, is mutated in Cornelia de Lange syndrome. Nat. Genet..

[3] Krantz I.D., McCallum J., DeScipio C., Kaur M., Gillis L.A., Yaeger D., Jukofsky L., Wasserman N., Bottani A., Morris C.A. (2004). Cornelia de Lange syndrome is caused by mutations in NIPBL, the human homolog of *Drosophila melanogaster* Nipped-B. Nat. Genet..

[4] Liu J., Feldman R., Zhang Z., Deardorff M.A., Haverfield E.V., Kaur M., Li J.R., Clark D., Kline A.D., Waggoner D.J. (2009). SMC1A expression and mechanism of pathogenicity in probands with X-linked Cornelia de Lange syndrome. Hum. Mutat..

[5] Musio A., Selicorni A., Focarelli M.L., Gervasini C., Milani D., Russo S., Vezzoni P., Larizza L. (2006). X-linked Cornelia de Lange syndrome owing to SMC1L1 mutations. Nat. Genet..

[6] Deardorff M.A., Kaur M., Yaeger D., Rampuria A., Korolev S., Pie J., Gil-Rodriguez C., Arnedo M., Loeys B., Kline A.D. (2007). Mutations in cohesin complex members SMC3 and SMC1A cause a mild variant of Cornelia de Lange syndrome with predominant mental retardation. Am. J. Hum. Genet..

[7] Borck G., Zarhrate M., Bonnefont J.P., Munnich A., Cormier-Daire V., Colleaux L. (2007). Incidence and clinical features of X-linked Cornelia de Lange syndrome due to SMC1L1 mutations. Hum. Mutat..

[8] Deardorff M.A., Wilde J.J., Albrecht M., Dickinson E., Tennstedt S., Braunholz D., Monnich M., Yan Y., Xu W., Gil-Rodriguez M.C. (2012). RAD21 mutations cause a human cohesinopathy. Am. J. Hum. Genet..

[9] Deardorff M.A., Bando M., Nakato R., Watrin E., Itoh T., Minamino M., Saitoh K., Komata M., Katou Y., Clark D. (2012). HDAC8 mutations in Cornelia de Lange syndrome affect the cohesin acetylation cycle. Nature.

[10] Nasmyth K., Haering C.H. (2009). Cohesin: its roles and mechanisms. Ann. Rev. Genet..

[11] Gillis L.A., McCallum J., Kaur M., DeScipio C., Yaeger D., Mariani A., Kline A.D., Li H.-h., Devoto M., Jackson L.G. (2004). NIPBL mutational analysis in 120 individuals with Cornelia de Lange syndrome and evaluation of genotype-phenotype correlations. Am. J. Hum. Genet..

[12] Bhuiyan Z.A., Klein M., Hammond P., van Haeringen A., Mannens M.M., Van Berckelaer-Onnes I., Hennekam R.C. (2006). Genotype-phenotype correlations of 39 patients with Cornelia De Lange syndrome: the Dutch experience. J. Med. Genet..

[13] Rohatgi S., Clark D., Kline A.D., Jackson L.G., Pie J., Siu V., Ramos F.J., Krantz I.D., Deardorff M.A. (2010). Facial diagnosis of mild and variant CdLS: insights from a dysmorphologist survey. Am. J. Med. Genet. Part A,.

[14] Ciosk R., Shirayama M., Shevchenko A., Tanaka T., Toth A., Nasmyth K. (2000). Cohesin's binding to chromosomes depends on a separate complex consisting of Scc2 and Scc4 proteins. Mol. Cell.

[15] Kawauchi S., Calof A.L., Santos R., Lopez-Burks M.E., Young C.M., Hoang M.P., Chua A., Lao T., Lechner M.S., Daniel J.A. (2009). Multiple organ system defects and transcriptional dysregulation in the Nipbl(+/-) mouse, a model of Cornelia de Lange syndrome. PLoS Genet..

[16] Castronovo P., Gervasini C., Cereda A., Masciadri M., Milani D., Russo S., Selicorni A., Larizza L. (2009). Premature chromatid separation is not a useful diagnostic marker for Cornelia de Lange syndrome. Chrom. Res..

[17] Schuldiner O., Berdnik D., Levy J.M., Wu J.S., Luginbuhl D., Gontang A.C., Luo L. (2008). piggyBac-based mosaic screen identifies a postmitotic function for cohesin in regulating developmental axon pruning. Dev. Cell.

[18] Pauli A., Althoff F., Oliveira R.A., Heidmann S., Schuldiner O., Lehner C.F., Dickson B.J., Nasmyth K. (2008). Cell-type-specific TEV protease cleavage reveals cohesin functions in *Drosophila* neurons. Dev. Cell.

[19] Seitan V.C., Hao B., Tachibana-Konwalski K., Lavagnolli T., Mira-Bontenbal H., Brown K.E., Teng G., Carroll T., Terry A., Horan K. (2011). A role for cohesin in T-cell-receptor rearrangement and thymocyte differentiation. Nature.

[20] Liu J., Zhang Z., Bando M., Itoh T., Deardorff M.A., Clark D., Kaur M., Tandy S., Kondoh T., Rappaport E. (2009). Transcriptional dysregulation in NIPBL and cohesin mutant human cells. PLoS Biol..

[21] Schaaf C.A., Kwak H., Koenig A., Misulovin Z., Gohara D.W., Watson A., Zhou Y., Lis J.T., Dorsett D. (2013). Genome-wide control of RNA polymerase II activity by cohesin. PLoS Genet..

[22] Dorsett D., Eissenberg J.C., Misulovin Z., Martens A., Redding B., McKim K. (2005). Effects of sister chromatid cohesion proteins on cut gene expression during wing development in *Drosophila*. Development.

[23] Rollins R.A., Morcillo P., Dorsett D. (1999). Nipped-B, a *Drosophila* homologue of chromosomal adherins, participates in activation by remote enhancers in the cut and Ultrabithorax genes. Genetics.

[24] Rollins R.A., Korom M., Aulner N., Martens A., Dorsett D. (2004). Drosophila nipped-B protein supports sister chromatid cohesion and opposes the stromalin/Scc3 cohesion factor to facilitate long-range activation of the cut gene. Mol. Cell. Biol..

[25] Parelho V., Hadjur S., Spivakov M., Leleu M., Sauer S., Gregson H.C., Jarmuz A., Canzonetta C., Webster Z., Nesterova T. (2008). Cohesins functionally associate with CTCF on mammalian chromosome arms. Cell.

[26] Rubio E.D., Reiss D.J., Welcsh P.L., Disteche C.M., Filippova G.N., Baliga N.S., Aebersold R., Ranish J.A., Krumm A. (2008). CTCF physically links cohesin to chromatin. Proc. Natl Acad. Sci. USA.

[27] Stedman W., Kang H., Lin S., Kissil J.L., Bartolomei M.S., Lieberman P.M. (2008). Cohesins localize with CTCF at the KSHV latency control region and at cellular c-myc and H19/Igf2 insulators. EMBO J..

[28] Wendt K.S., Yoshida K., Itoh T., Bando M., Koch B., Schirghuber E., Tsutsumi S., Nagae G., Ishihara K., Mishiro T. (2008). Cohesin mediates transcriptional insulation by CCCTC-binding factor. Nature.

[29] Hadjur S., Williams L.M., Ryan N.K., Cobb B.S., Sexton T., Fraser P., Fisher A.G., Merkenschlager M. (2009). Cohesins form chromosomal cis-interactions at the developmentally regulated IFNG locus. Nature.

[30] Mishiro T., Ishihara K., Hino S., Tsutsumi S., Aburatani H., Shirahige K., Kinoshita Y., Nakao M. (2009). Architectural roles of multiple chromatin insulators at the human apolipoprotein gene cluster. EMBO J..

[31] Nativio R., Wendt K.S., Ito Y., Huddleston J.E., Uribe-Lewis S., Woodfine K., Krueger C., Reik W., Peters J.M., Murrell A. (2009). Cohesin is required for higher-order chromatin conformation at the imprinted IGF2-H19 locus. PLoS Genet..

[32] Degner S.C., Verma-Gaur J., Wong T.P., Bossen C., Iverson G.M., Torkamani A., Vettermann C., Lin Y.C., Ju Z., Schulz D. (2011). CCCTC-binding factor (CTCF) and cohesin influence the genomic architecture of the Igh locus and antisense transcription in pro-B cells. Proc. Natl Acad. Sci. USA.

[33] Chien R., Zeng W., Kawauchi S., Bender M.A., Santos R., Gregson H.C., Schmiesing J.A., Newkirk D., Kong X., Ball A.R. (2011). Cohesin mediates chromatin interactions that regulate mammalian β-globin expression. J. Biol. Chem..

[34] Kim Y.J., Cecchini K.R., Kim T.H. (2011). Conserved, developmentally regulated mechanism couples chromosomal looping and heterochromatin barrier activity at the homeobox gene A locus. Proc. Natl Acad. Sci. USA.

[35] Seitan V.C., Merkenschlager M. (2012). Cohesin and chromatin organisation. Curr. Opin. Genet. Dev..

[36] Kagey M.H., Newman J.J., Bilodeau S., Zhan Y., Orlando D.A., van Berkum N.L., Ebmeier C.C., Goossens J., Rahl P.B., Levine S.S. (2010). Mediator and cohesin connect gene expression and chromatin architecture. Nature.

[37] Schmidt D., Schwalie P.C., Ross-Innes C.S., Hurtado A., Brown G.D., Carroll J.S., Flicek P., Odom D.T. (2010). A CTCF-independent role for cohesin in tissue-specific transcription. Genome Res..

[38] Eskeland R., Leeb M., Grimes G.R., Kress C., Boyle S., Sproul D., Gilbert N., Fan Y., Skoultchi A.I., Wutz A. (2010). Ring1B compacts chromatin structure and represses gene expression independent of histone ubiquitination. Mol. Cell.

[39] Gurdon J.B. (1976). Injected nuclei in frog oocytes: fate, enlargement, and chromatin dispersal. J. Embryol. Exp. Morphol..

[40] Shen X., Yu L., Weir J.W., Gorovsky M.A. (1995). Linker histones are not essential and affect chromatin condensation in vivo. Cell.

[41] Mazumder A., Roopa T., Basu A., Mahadevan L., Shivashankar G.V. (2008). Dynamics of chromatin decondensation reveals the structural integrity of a mechanically prestressed nucleus. Biophys. J..

[42] Pehlivan D., Hullings M., Carvalho C.M., Gonzaga-Jauregui C.G., Loy E., Jackson L.G., Krantz I.D., Deardorff M.A., Lupski J.R. (2012). NIPBL rearrangements in Cornelia de Lange syndrome: evidence for replicative mechanism and genotype-phenotype correlation. Genet. Med..

[43] Pie J., Gil-Rodriguez M.C., Ciero M., Lopez-Vinas E., Ribate M.P., Arnedo M., Deardorff M.A., Puisac B., Legarreta J., de Karam J.C. (2010). Mutations and variants in the cohesion factor genes NIPBL, SMC1A, and SMC3 in a cohort of 30 unrelated patients with Cornelia de Lange syndrome. Am. J. Med. Genet. A.

[44] Oka Y., Suzuki K., Yamauchi M., Mitsutake N., Yamashita S. (2011). Recruitment of the cohesin loading factor NIPBL to DNA double-strand breaks depends on MDC1, RNF168 and HP1gamma in human cells. Biochem. Biophys. Re.s Commun..

[45] Neuwald A.F., Hirano T. (2000). HEAT repeats associated with condensins, cohesins, and other complexes involved in chromosome-related functions. Genome Res..

[46] Jahnke P., Xu W., Wülling M., Albrecht M., Gabriel H., Gillessen-Kaesbach G., Kaiser F.J. (2008). The cohesin loading factor NIPBL recruits histone deacetylases to mediate local chromatin modifications. Nucleic Acids Res..

[47] Caron H., van Schaik B., van der Mee M., Baas F., Riggins G., van Sluis P., Hermus M.C., van Asperen R., Boon K., Voute P.A. (2001). The human transcriptome map: clustering of highly expressed genes in chromosomal domains. Science.

[48] The ENCODE consortium (2012). An integrated encyclopedia of DNA elements in the human genome. Nature.

[49] Cheung V.G., Spielman R.S., Ewens K.G., Weber T.M., Morley M., Burdick J.T. (2005). Mapping determinants of human gene expression by regional and genome-wide association. Nature.

[50] Barski A., Cuddapah S., Cui K., Roh T.Y., Schones D.E., Wang Z., Wei G., Chepelev I., Zhao K. (2007). High-resolution profiling of histone methylations in the human genome. Cell.

[51] Vavouri T., Lehner B. (2012). Human genes with CpG island promoters have a distinct transcription-associated chromatin organization. Genome Biol..

[52] Yokota H., Singer M.J., van den Engh G.J., Trask B.J. (1997). Regional differences in the compaction of chromatin in human G0/G1 interphase nuclei. Chrom. Res..

[53] Gilbert N., Boyle S., Fiegler H., Woodfine K., Carter N.P., Bickmore W.A. (2004). Chromatin architecture of the human genome: gene-rich domains are enriched in open chromatin fibers. Cell.

[54] Chambeyron S., Bickmore W.A. (2004). Chromatin decondensation and nuclear reorganization of the HoxB locus upon induction of transcription. Genes Dev..

[55] Morey C., Da Silva N.R., Perry P., Bickmore W.A. (2007). Nuclear reorganisation and chromatin decondensation are conserved, but distinct, mechanisms linked to Hox gene activation. Development.

[56] Guo Y., Monahan K., Wu H., Gertz J., Varley K.E., Li W., Myers R.M., Maniatis T., Wu Q. (2012). CTCF/cohesin-mediated DNA looping is required for protocadherin alpha promoter choice. Proc. Natl Acad. Sci. USA.

[57] Junier I., Dale R.K., Hou C., Kepes F., Dean A. (2012). CTCF-mediated transcriptional regulation through cell type-specific chromosome organization in the beta-globin locus. Nucleic Acids Res..

[58] Gard S., Light W., Xiong B., Bose T., McNairn A.J., Harris B., Fleharty B., Seidel C., Brickner J.H., Gerton J.L. (2009). Cohesinopathy mutations disrupt the subnuclear organization of chromatin. J. Cell Biol..

[59] Monahan K., Rudnick N.D., Kehayova P.D., Pauli F., Newberry K.M., Myers R.M., Maniatis T. (2012). Role of CCCTC binding factor (CTCF) and cohesin in the generation of single-cell diversity of Protocadherin-alpha gene expression. Proc. Natl Acad. Sci. USA.

[60] Muto A., Calof A.L., Lander A.D., Schilling T.F. (2011). Multifactorial origins of heart and gut defects in nipbl-deficient zebrafish, a model of Cornelia de Lange syndrome. PLoS Biol..

[61] Rasheed S., Nelson-Rees W.A., Toth E.M., Arnstein P., Gardner M.B. (1974). Characterization of a newly derived human sarcoma cell line (HT-1080). Cancer.

[62] Gerth-Kahlert C., Williamson K., Ansari M., Rainger J.K., Hingst V., Zimmermann T., Tech T., Guthoff R.F., van Heyningen V., FitzPatrick D.R. (2013). Clinical and mutation analysis of 51 probands with anophthalmia and/or severe microphthalmia from a single center. Mol. Genet. Genomic Med..

