# Supplementary material for: Regional chromatin decompaction in Cornelia de Lange syndrome associated with NIPBL disruption can be uncoupled from cohesin and CTCF
Source: Hum Mol Genet. 2013 Jun 10;22(20):4180–93. doi: 10.1093/hmg/ddt265 (PMC3781641; doi:10.1093/hmg/ddt265)
Supplement: Supplementary Data [file supp_22_20_4180__index.html]

Regional chromatin decompaction in Cornelia de Lange syndrome associated with NIPBL disruption can be uncoupled from cohesin and CTCF — Regional chromatin decompaction in Cornelia de Lange syndrome associated with NIPBL disruption can be uncoupled from cohesin and CTCF — Supplementary Data 

# Regional chromatin decompaction in Cornelia de Lange syndrome associated with NIPBL disruption can be uncoupled from cohesin and CTCF

## 

Supplementary Data

**Files in this Data Supplement:**

- Supplementary Data - eps format
